# Clinical Efficacy and Safety of the Herbal Prescription, HH333, in Preventing Recurrent Stroke in Patients With Ischemic Stroke Induced by Small-Vessel Disease: Protocol for Multicenter, Double-Blind, Randomized, Prospective, Pilot Clinical Trial

**DOI:** 10.2196/70953

**Published:** 2025-05-13

**Authors:** Han-Gyul Lee, Seungwon Kwon, Woo-Sang Jung, Sang-Kwan Moon, Cheol-Hyun Kim, Dong-Jun Choi

**Affiliations:** 1 Department of Cardiology and Neurology College of Korean Medicine, Kyung Hee University Medical Center Kyung Hee University Seoul Republic of Korea; 2 Department of Internal Medicine and Neuroscience College of Korean Medicine, Wonkwang University Gwangju Medical Center Wonkwang University Gwangju Republic of Korea; 3 Department of Internal Medicine Dongguk University Ilsan Oriental Hospital Ilsan Republic of Korea

**Keywords:** HH333, ischemic stroke, herbal medicine, randomized controlled trial, protocol

## Abstract

**Background:**

Patients with ischemic stroke are at high risk of recurrence, making preventive care an important factor. Current antiplatelet therapy for recurrence prevention treatment has several limitations. Recent retrospective observational studies suggested that HH333, an herbal prescription, has an inhibitory effect on stroke recurrence in small-vessel diseases.

**Objective:**

This study aims to propose a protocol for evaluating the efficacy and safety of HH333 in patients with ischemic stroke induced by small-vessel disease.

**Methods:**

In this multicenter, double-blind, randomized, prospective, pilot clinical trial, 236 patients from 3 university Korean medicine hospitals in South Korea with ischemic stroke caused by small-vessel disease will be recruited and randomly assigned to either the HH333 or the placebo group. Both patients and investigators will be blinded to prevent access to the allocation results. The HH333 group will take 2 capsules of HH333 once daily for 720 days, whereas the placebo group will take HH333 placebo capsules in the same manner. Efficacy will be assessed using the recurrence rate of ischemic stroke, which will be assessed on days 30, 90, 180, 270, 360, 450, 540, 630, 720, and 750 after starting the medication. The effects on quality of life and fatigue with the Fatigue Severity Scale (FSS), Fatigue Assessment Scale (FAS), and Korean Patient Health Questionnaire (K-PHQ-9), functional improvement with Korean National Institutes of Health Stroke Scale (K-NIHSS), modified Rankin Scale (mRS), Korean modified Barthel Index (K-mBI), and Korean Montreal Cognitive Assessment (K-MoCA) and Pattern Identification also will be evaluated on days 0, 90, 180, 270, 360, 450, 540, 630, and 720 after starting the medication. Safety will be evaluated by performing blood and urine tests and electrocardiography on days 30, 90, 180, 270, 360, 450, 540, 630, and 720 after starting the medication.

**Results:**

Recruitment for the study started on May 22, 2024, and is scheduled to end on November 30, 2026. As of November 13, 2024, a total of 12 participants have been randomized.

**Conclusions:**

The protocol will provide a detailed process for a clinical trial evaluating the efficacy of preventing recurrent ischemic stroke caused by small-vessel disease and improving neurologic symptoms and the safety of HH333 in ischemic stroke. The results of this study provide a basis for alternative treatments to prevent and treat ischemic stroke.

**Trial Registration:**

Clinical Research Information Service KCT0009431; https://tinyurl.com/y2ctvje8

**International Registered Report Identifier (IRRID):**

DERR1-10.2196/70953

## Introduction

Patients with ischemic stroke are at high risk for recurrence [1]. Generally, ischemic stroke recurrence rates over 5 years range from 10% to 53% [2]. Recurrent stroke is associated with twice the risk of death and worse functional outcomes compared to patients with first-time stroke [3]. It also costs twice as much as hospitalization for a first-time stroke [4].

Antiplatelet or anticoagulant therapy is currently recommended as the first-line treatment for the prevention of recurrent ischemic stroke [5]. However, there are differences in the pathogenesis of ischemic stroke between Western and Asian populations, with Northeast Asians such as Koreans, Chinese, and Japanese having a higher proportion of strokes caused by small-vessel disease than Westerners [6,7]. Ischemic stroke induced by small-vessel disease usually results from the gradual thickening of the vessel wall caused by hypertension and is more likely to be accompanied by intracerebral microbleeds [8]. This may limit the use of antiplatelet or anticoagulant medications that may increase the bleeding propensity. HH333 is a complex herbal medicine that has inhibitory effects on coenzyme A reductase and pancreatic lipase [9], antioxidant and anti-inflammatory effects through suppression of nitric oxide biosynthesis and prostaglandin E2 [10], antihyperlipidemic and anti-inflammatory effects through activation of nitric oxide production [11], and endothelial cell proliferation through activation of MAP kinase [12]. Previous clinical studies have reported antihypertensive effects in stroke patients with hypertension [13], antilipidemic effects in patients with hypercholesterolemia [14], immediately improving cerebral blood flow in normal subjects [15], and improvement in arterial stiffness in patients with increased pulse wave velocity [16]. Based on these effects, HH333 is a promising medication for cerebrovascular and cardiovascular disease [17].

Specifically, HH333 inhibits the progression of microangiopathy, a key factor in small-vessel disease, and thus prevents the recurrence of ischemic stroke induced by small-vessel disease with a 2-year recurrence rate was 1.12%-2.02% [18-20], which is less than conventional antiplatelet agents [21-24]. Regarding safety, a previous retrospective study showed that the incidence of adverse events with HH333 was 2.0%, with no serious adverse events (SAEs) [25]. However, since these studies were all single-arm retrospective observational studies, there is no comparative analysis with a control group, and the data rely on medical records, which can be inaccurate. Therefore, there is a need for prospective clinical studies with a control group to rationalize and better understand these findings.

We present a protocol for a randomized controlled clinical trial to validate the clinical efficacy and safety of HH333 in patients with ischemic stroke induced by small-vessel disease. This study aimed to provide a high level of evidence for the efficacy of HH333 in ischemic stroke and ultimately propose a novel alternative for stroke treatment.

## Methods

### Study Design

**Figure 1:**
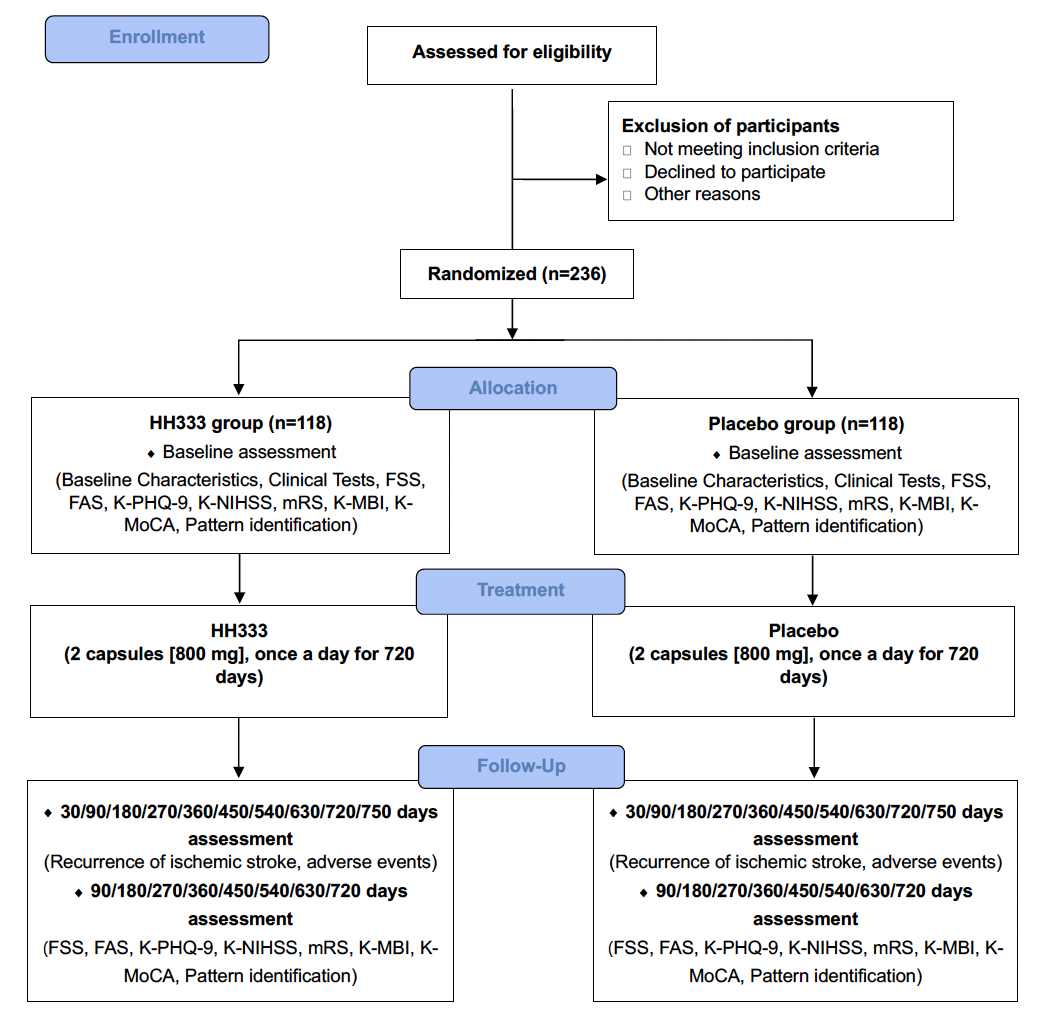
Flowchart of the study process FSS, Fatigue Severity Scale; FAS, Fatigue Assessment Scale; K-mBI, Korean modified Barthel Index; K-MoCA, Korean Montreal Cognitive Assessment; K-NIHSS, Korean National Institutes of Health Stroke Scale; K-PHQ-9, Korean Patient Health Questionnaire; mRS, modified Rankin Scale.

This multicenter, double-blind, randomized controlled trial (RCT) aimed to assess the prevention of recurrent ischemic stroke and the safety of HH333 in patients with ischemic stroke induced by small-vessel disease. It was conducted at 3 university Korean medicine hospitals in South Korea: Kyung Hee University Korean Medicine Hospital, Dongguk University Ilsan Korean Medicine Hospital, Wonkwang University Gwangju Korean Medicine Hospital. Within 90 days of onset at baseline, patients will receive HH333 or a placebo, 2 capsules (800 mg) at a time, once a day for 720 days. The clinical effect of HH333 will be evaluated by ischemic stroke recurrence, which is the primary efficacy endpoint, at baseline and on days 30, 90, 180, 270, 360, 450, 540, 630, 720, and 750 after starting the medication. Total scores on the Fatigue Severity Scale (FSS), Fatigue Assessment Scale (FAS), Korean Patient Health Questionnaire (K-PHQ-9), and the probability of each type of Pattern Identification (fire heat, dampness-phlegm, qi deficiency or yin deficiency) will be evaluated as secondary efficacy endpoints at baseline and 90, 180, 270, 360, 450, 540, 630, and 720 days after starting medication. The total scores on the Korean National Institutes of Health Stroke Scale (K-NIHSS), modified Rankin Scale (mRS), Korean Modified Barthel Index (K-mBI), and Korean Montreal Cognitive Assessment (K-MoCA) will also be measured as exploratory endpoints at the same time as the secondary efficacy endpoints. The safety of HH333 will be assessed based on the presence of adverse events at the same time as the primary efficacy endpoints. The enrollment, interventions, and assessments of the study are summarized in Table 1. A schematic of the study process is shown in Figure 1.

**Table 1:** Schedule of enrollment, interventions, and assessments.

Visit	0	1	2	3	4	5	6	7	8	9	10	11^a^	UV^b^
Day	–14^c^	0	25-35	80-100	165-195	255-285	345-375	435-465	525-555	615-645	705-735	745-755	
Informed consent	✓												
Demographic information^d^	✓												
Medical history^e^	✓												
History of ischemic stroke^f^	✓												
Medications^g^	✓												
Physical examination	✓	✓	✓	✓	✓	✓	✓	✓	✓	✓	✓		If needed
Vital sign	✓	✓	✓	✓	✓	✓	✓	✓	✓	✓	✓		If needed
Physical measurements	✓												
Laboratory tests^h^	✓												
Electrocardiogram	✓		✓	✓	✓	✓	✓	✓	✓	✓	✓		If needed
Evaluating inclusion and exclusion criteria	✓												
Randomized allocation		✓											
Experiment medication administration		✓	✓	✓	✓	✓	✓	✓	✓	✓	✓		
Medication adherence^i^			✓	✓	✓	✓	✓	✓	✓	✓	✓		If needed
Concomitant treatment		✓	✓	✓	✓	✓	✓	✓	✓	✓	✓	✓	If needed
Primary efficacy endpoint: recurrence of ischemic stroke			✓	✓	✓	✓	✓	✓	✓	✓	✓	✓	If needed
Secondary efficacy endpoints^j^		✓		✓	✓	✓	✓	✓	✓	✓	✓		If needed
Exploratory endpoints^k^		✓		✓	✓	✓	✓	✓	✓	✓	✓		If needed
Safety assessments^l^			✓	✓	✓	✓	✓	✓	✓	✓	✓		If needed
Adverse events^m^		✓	✓	✓	✓	✓	✓	✓	✓	✓	✓	✓	

^a^Telephone investigation.

^b^UV: Unscheduled visit. Visiting on a day other than the original visit schedule due to an adverse event, etc.

^c^Screening performed within 14 days of informed consent.

^d^Age, sex, smoking, and alcohol history.

^e^Within 1 year prior to visit 0.

^f^Time of first onset and diagnosis (including year, month, and day if possible), date of computed tomography or magnetic resonance imaging, ischemic stroke territory, Trial of Org 10172 in Acute Stroke Treatment classification, history of stroke-related surgery, stroke risk factors, and stroke-related symptoms.

^g^Within 4 weeks prior to visit 0.

^h^Complete blood count (red blood cell, hemoglobin, hematocrit, platelet, white blood cell); biochemistry (fasting blood glucose, blood urea nitrogen, creatinine, natrium, potassium, chloride, aspartate aminotransferase, alanine aminotransferase, C-reactive protein, calcium, uric acid); urine human chorionic gonadotropin for women of childbearing age, excluding sterilization and menopause (more than 12 months since last menstrual period); premature menopause before the age of 40 years, follicle-stimulating hormone test for differential diagnosis.

^i^Drop out if <80%.

^j^Total scores of Fatigue Severity Scale (FSS), Fatigue Assessment Scale (FAS), Korean Patient Health Questionnaire (K-PHQ-9), and probability of each type of Pattern Identification (fire heat, dampness-phlegm, Qi deficiency, and Yin deficiency).

^k^Total scores of the Korean National Institutes of Health Stroke Scale (K-NIHSS), Korean modified Rankin Scale (K-mRS), Korean modified Barthel Index (K-mBI), Korean Montreal Cognitive Assessment (K-MoCA).

^l^Complete blood count (red blood cell, hemoglobin, hematocrit, platelet, white blood cell); biochemistry (fasting blood glucose, blood urea nitrogen, creatinine, natrium, potassium, chloride, aspartate aminotransferase, alanine aminotransferase, C-reactive protein, calcium, uric acid); urine tests (specific gravity, pH, albumin or protein, glucose, ketone, bilirubin, occult blood, urobilinogen, nitrite, leukocyte, human chorionic gonadotropin for women of childbearing age); electrocardiogram; hemorrhage events (cerebral hemorrhage, gastrointestinal bleeding, etc) related to antiplatelet or anticoagulant; vital sign; physical examination.

^m^Self-reported by the participant or judged by the investigator.

### Eligibility

Eligibility criteria and other details are listed in Textbox 1.

**Inclusion criteria, exclusion criteria, and dropout criteria.**

**Inclusion criteria**

- Men and women aged 19 to 80 years.
- Diagnosed with ischemic stroke induced by small-vessel disease in the Trial of Org 10172 in Acute Stroke Treatment (TOAST) classification within 90 days of screening based on new neurologic and brain imaging (computed tomography or magnetic resonance) findings to support them.
- Participants had no communication problems and voluntarily agreed to participate.

**Exclusion criteria**

- Diagnosis of degenerative brain diseases (eg, Parkinson’s disease, Alzheimer disease, etc).
- Have a brain condition other than ischemic stroke (eg, brain tumor, traumatic brain injury, arteriovenous malformation, Moyamoya disease, and stroke due to these conditions).
- Unstable vital signs at screening (eg, systolic blood pressure greater than 160 mm Hg despite taking blood pressure medications).
- Serious medical condition (eg, liver cirrhosis, chronic kidney disease stage V, or severe heart failure of New York Heart Association (NYHA) grade III or greater).
- History of malignancy, including leukemia and lymphoma, within the past 5 years.
- Hepatic and renal dysfunctions.
  Aspartate aminotransferase, alanine aminotransferase ≥2 times the upper limit of normal at screening.
  An estimated glomerular filtration rate < 60 mL/min/1.73 m2 at screening.
- Within 24 hours of onset.
- Patients with Korean National Institutes of Health Stroke Scale (K-NIHSS) score 21 to 42.
- Pregnant, nurses, or women of childbearing potential who have negative pregnancy test results and have not agreed to use contraception during the study.
- Previously experienced hypersensitivity after taking HH333’s constituent medicines *Scutellariae radix*, *Coptidis rhizoma*, *Phellodendri cortex*, *Gardeniae fructus*, and *Rhei rhizoma*.
- Any other study medications administered within 30 days prior to screening.
- Patients who have not been administered the study medication or who have participated in a simple observational clinical trial are eligible.
- Difficulty taking the doses required in this study.
- Judged by the investigator as nonconformity for participation in the trial for any reason.
- Plans to use anticoagulants during the study or have difficulty discontinuing them.

**Dropout criteria**

If the researcher determines that it is not appropriate for the study to continue according to the dropout criteria, the researcher may stop the treatment and evaluation and drop the participant. Participants may also drop out of the study at any time of their free will. The dropout criteria were as follows:

- Found not to meet inclusion or exclusion criteria after screening.
- Patients or caregivers withdraw consent during the study period.
- Patients or caregivers request to discontinue the study or refuse treatment during the study.
- Participants cannot be traced.
- According to the judgment of the investigator, the patient is no longer able to participate in the study because of adverse events or comorbidities.
- Study medication compliance <80%.
- Participation in another clinical trial.
- Judged by the investigator as unfit to continue the study for any other reason.

### Recruitment

A total of 236 patients with ischemic stroke induced by small-vessel disease will be recruited through walk-in or hospital and school bulletin boards, public transportation billboards, newsletters, and other online advertisements. Participants who wish to participate will receive the consent form and provide written consent of their own free will. Multimedia Appendix 1 presents the research consent form. Participating in the study will be decided if the researcher determines that they are suitable through the screening process. The screening items were as follows: demographic information, medical history, history of ischemic stroke, medications, physical examination, vital signs, physical measurements, laboratory findings, electrocardiogram, and evaluation of the inclusion and exclusion criteria. All data were simultaneously recorded in a case report form.

### Randomization, Blinding, and Allocation Concealment

A total of 236 participants who met the criteria were assigned to the HH333 or control group at a ratio of 1:1 by a statistician independent of this clinical study, using the block randomization method with R 4.4.0 (Comprehensive R Archive Network). Patients and investigators will be blinded to prevent access to the allocation results. Patients will be blinded to the study medication they are administered because they are taking either the treatment medication or a placebo. The treatment medication and placebo will be identical in size, shape, and appearance and packaged in the same type of packaging.

For allocation concealment, an independent statistician who generates the random numbers delivers a randomization code to each clinical trial site in a sealed, impermeable envelope. The investigators at each site will open the randomization envelopes in sequence in front of the patients to assign participants, and the open envelopes will be kept separate.

### Interventions

HH333 or placebo capsules (one dose: 2 capsules, 800 mg) will be orally administered with water once a day on an empty stomach after waking for 720 days. HH333 is an herbal medicine manufactured by Kyungjin Pharmaceutical Co, Ltd. (Icheon, Republic of Korea). It is a capsule formulation derived from 80% ethanol extracts of *Scutellariae radix, Coptidis rhizoma, Phellodendri cortex, Gardeniae fructus*, and *Rhei rhizoma* (Table 2). The placebo was a capsule manufactured by Kyungjin Pharmaceutical Co., Ltd. (Icheon, Republic of Korea) with the same appearance as HH333.

**Table 2:** Composition of HH333.

Constituent herbs	Scientific names	Weight (g)	
*Scutellariae radix*	*Scutellaria baicalensis* Georgi (from Republic of Korea)	0.28	
*Coptidis rhizoma*	*Coptis japonica* Makino (from Republic of Korea)	0.28	
*Phellodendri cortex*	*Phellodendron amurense* Ruprecht (from Republic of Korea)	0.28	
*Gardenia fructus*	*Gardenia jasminodes* Ellis (from Republic of Korea)	0.28	
*Rhei rhizoma*	*Rheum palmatum* L. (from Republic of Korea)	0.07	
Total			1.2

Medication compliance will be recorded in patients taking either HH333 or placebo. The participants will be instructed to return the empty containers of study medications taken and the remaining medications at each visit to count the actual number of doses of study medications. Compliance was calculated as follows:

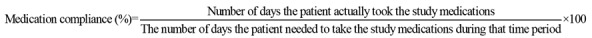



If compliance was less than 80% at any point during the study, the participant was excluded from the study

### Outcome Measurements

The clinical efficacy of HH333 in patients with ischemic stroke induced by small-vessel disease will be evaluated based on whether ischemic stroke recurrence occurs after administration of HH333. Furthermore, the effects on quality of life and fatigue will be evaluated by measuring FSS, FAS, and K-PHQ-9 scores, and changes in pattern identification probability will be investigated to provide basic data for the Korean medicine treatment of stroke. The K-NIHSS, mRS, K-mBI, and K-MoCA will be used to assess functional improvements related to ischemic stroke. All outcome measurements will be conducted through face-to-face visits.

### Primary Endpoint: Recurrent Ischemic Stroke

Recurrent ischemic stroke within 750 days while taking the study medication was investigated. Recurrent ischemic stroke is defined as a new neurological finding supported by new imaging findings. New neurological findings corresponded to the sudden onset of neurological symptoms identified on neurological examinations that did not occur prior to study participation. New radiological findings are abnormal findings on brain imaging (computed tomography or magnetic resonance), which can explain new neurological findings that did not exist before study participation. The investigation will be conducted on days 30, 90, 180, 270, 360, 450, 540, 630, 720, and day 750, which is 30 days after the end of administration.

### Secondary Endpoints: FSS, FAS, K-PHQ-9, Pattern Identification

The FSS is a 9-item, self-reported screening tool developed by Krupp et al [26] that assesses fatigue over the past week on a scale of 1 to 7 per item, with higher total scores indicating higher levels of fatigue. The FAS is a 10-item, self-reported screening tool developed by Michielsen et al [27] that examines the psychometric qualities due to fatigue on a scale from 1=“Never” to 5=“Always” per item, with a higher total score indicating higher levels of the psychological impact of fatigue. The PHQ-9, developed by Kroenke et al [28] was validated in the Korean version [29]. It comprises 9 items in total. Respondents rate the frequency of symptoms on a scale of 0-3 per item, with a total score of 10 or higher indicating major depression. These secondary endpoints will assess the clinical effectiveness of HH333 on poststroke fatigue and depression. Pattern identification is a unique diagnostic system in Korean medicine, and the diagnosis of stroke is based on 4 patterns which are fire heat, Dampness-phlegm, Qi deficiency, and Yin deficiency [30]. Jung et al [31] developed an equation model that predicts the probability of each pattern identification diagnosis given variables for stroke pattern identification and is being used for Korean medicine's standardized diagnosis of patients with stroke. Using a stroke pattern identification prediction model, the change in each pattern identified after HH333 administration was collected to obtain data for stroke treatment in Korean medicine. Investigations were conducted during the administration period on days 0 (baseline), 90, 180, 270, 360, 450, 540, 630, and 720.

### Exploratory Endpoints: K-NIHSS, mRS, K-mBI, and K-MoCA

The NIHSS is a tool for assessing neurological deficits resulting from stroke developed by the National Institutes of Health [32] and is currently the most widely used tool for stroke severity assessment, with a Korean version validated and used [33]. It comprises 15 questions in 11 checkpoints, each of which is scored on a scale of 0 to 4, with a total score calculated, with higher scores indicating higher severity. The mRS is a tool developed by van Swieten et al to assess the degree of disability in patients with stroke [34] and is currently the most widely used stroke functional assessment scale [35]. It is rated on a scale of 0 to 6, with higher scores indicating more severe impairment. The mBI is a tool to assess independence in activities of daily living after stroke developed by Shah et al [36], and translated with validation in Korean [37]. It contains 10 questions, each rated on a scale of 0 to 5, 0 to 10, or 0 to 15, with a higher total score indicating a lower dependency. The MoCA is a tool that examines cognitive function and was originally developed to identify mild cognitive impairment [38] but is now widely used for cognitive impairment caused by stroke, and the Korean version has been validated and used [39]. It consists of visuospatial and executive (5 points), naming (2 points), attention (6 points), language (3 points), abstraction (2 points), delayed recall (5 points) and orientation (6 points). The perfect score is 30, with a higher total score indicating higher cognitive function. Investigations were conducted during the administration period on days 0 (baseline), 90, 180, 270, 360, 450, 540, 630, and 720.

### Safety Assessments and Monitoring

At each visit, participants will be screened for adverse events, laboratory tests, urine findings, electrocardiograms, hemorrhagic events such as cerebral hemorrhage or gastrointestinal bleeding related to antiplatelet or anticoagulant use, vital signs, and physical examinations. Laboratory findings included complete blood count (red blood cells, hemoglobin, hematocrit, platelets, and white blood cells) and biochemistry (fasting blood sugar, blood urea nitrogen, creatinine, natrium, potassium, chloride, aspartate aminotransferase, alanine aminotransferase, C-reactive protein, calcium, and uric acid). Urine findings included specific gravity, pH, albumin or protein, glucose, ketone, bilirubin, occult blood, urobilinogen, nitrite, leukocytes, and human chorionic gonadotropin in women of childbearing age. Adverse events included both self-reported symptoms and those judged by the investigator and were collected until 30 days after the end of administration.

After collecting all adverse events that appeared as test findings and symptoms in each group, we compared the frequency and severity of adverse events between the groups and their relationship with the study medications. Adverse events that are not related to the study medications do not meet the criteria for dropout but allow the patient to receive the best possible care regardless of the study. If SAEs occur, the study will be stopped immediately, in part or in whole, and the study researcher will report it to the principal investigator and the institutional review board for appropriate action.

### Concomitant Medication

All concomitant medications except conventional treatments for ischemic stroke are prohibited. Medications that have been used for the participants’ underlying medical condition for 12 weeks prior to screening that are considered stable and unlikely to affect the interpretation of the results of this study with no change in dosage or dose will be allowed with a note of the reason, as determined by the investigator. Transient symptoms unrelated to stroke, such as cold, flu, dyspepsia, constipation, and concomitant use, will be allowed on a one-time basis at the discretion of the investigator. The use of supplements such as vitamins is not permitted. The use of vitamin K antagonists, direct thrombin inhibitors, factor X inhibitors, and heparin (except for temporary use during percutaneous coronary intervention) that may affect bleeding propensity will be strictly prohibited.

### Sample Size

In previous studies, ischemic stroke recurrence rates of 12.5%-12.8% over 24 months with conventional antiplatelet therapy (aspirin or dipyridamole) [22] and 1.7% over the same period with HH333 [19] were reported. Therefore, the sample size was 106 patients per group and 212 patients in total at 2 years with a control group (P_2_=0.125-0.128), HH333 group (P_1_=0.017), a significance level (α) of 5% and a target power of 80%. Finally, a dropout rate of 10% was considered, resulting in a sample size of 118 patients per group for a total of 236 patients.

### Statistical Analysis

All statistical analyses were two-sided with a 5% significance level. The statistical program SAS (version 9.4 or more, SAS Institute) was used for the analysis. If at any point there are missing values or participants are dropped before the end of the study, the data will be treated as missing data. All data obtained from the participants were analyzed in 3 forms: full analysis set, per-protocol set, and safety set. Efficacy analysis will be conducted using the results of the full analysis set as the main analysis, and a per-protocol set analysis will be conducted separately to evaluate whether there are differences from the full analysis set results. Safety analysis was performed on the safety set and the safety of the study medications was evaluated. The full analysis set includes all data from participants with at least one efficacy assessment at the primary outcome after taking the study medication. The per-protocol set included participants who were eligible for full analysis and successfully completed the study. The safety set included all data from participants who received at least one dose of the study medication.

### Demographic Information Analysis

For each group, continuous data yielded the mean, SD, median, minimum, maximum, and 95% CIs, whereas categorical data yielded frequencies and percentages. To compare groups, we used a two-sample *t* test or Wilcoxon rank-sum test for continuous data, and a chi-square test or Fisher’s exact test for categorical data. Baseline information that was recognized as significantly different between the 2 groups was considered a covariate in the efficacy analysis to correct for heterogeneity between the treatment groups.

### Efficacy Analysis

#### Analysis of Primary Efficacy Endpoints

The number of events will be presented for ischemic stroke recurrence during the 2-year study medication treatment period, the odds ratio of stroke recurrence will be calculated, and logistic regression analysis will be performed with ischemic stroke recurrence as the dependent variable and other factors such as HH333 treatment, risk factors, and neurological deficits as independent variables.

#### Analysis of Secondary Efficacy and Exploratory Endpoints

Secondary efficacy endpoints that FSS, FAS, K-PHQ-9 total score change, each pattern identification probability change, and exploratory endpoints K-NIHSS, mRS, K-mBI, K-MoCA total score change are continuous variables, and the average for each measurement, SD, median, minimum value, maximum value will be presented and the difference between the HH333 group and the control group will be tested using repeated measures ANOVA.

### Safety Analysis

An adverse event is a new occurrence of a symptom after study medication administration that was not observed before administration and includes unintended consequences such as abnormal laboratory findings, symptoms, and temporary events related to the study medications. The severity of adverse events, both self-reported and non–self-reported, will be assessed by the investigator by applying Spilker’s classification as follows: mild, not requiring additional intervention, nor significantly inhibiting the normal lifestyle (function) of the participant; moderate, significantly inhibiting the normal lifestyle (function) of the participant and may need additional intervention, recovering afterward; severe, severe adverse events requiring intensive intervention; and leaving sequelae. The causal relationship between adverse events and the study medication will be assessed by the investigator using the following six-level grading scale: (1) definitely related, (2) possibly related, (3) probably related, (4) not related, (5) definitely not related, and (6) unknown.

For all adverse events and abnormal findings on examination, the symptoms, frequency, and degree of relationship with the study medication were recorded in detail for each group. The number and proportion of participants in whom adverse events occurred at least once, 95% CI, and number of occurrences within each group will be presented. Differences in proportions between groups will be tested using the chi-square test or Fisher exact test.

### Ethical Considerations

This study protocol was approved by the Kyung Hee University Korean Medicine Hospital institutional review board (KOMCIRB 2023-05-010-007; approved on April 19, 2024), and conducted in accordance with the SPIRIT (Standard Protocol Items: Recommendations for Interventional Trials) checklist. Multimedia Appendix 2 presents the SPIRIT checklist of the study protocol [40]. Participants who wish to participate receive an explanation of the consent form and give written consent of their own free will. All participants receive cash compensation of ₩50,000 per visit. In addition, all costs related to the clinical trial medications and examinations are covered by the research team. All data from participants will be simultaneously recorded in an electronic case report form with an identification code rather than the participant’s name to protect personal information.

## Results

The study was funded by the Ministry of Health and Welfare, Republic of Korea (RS-2022-KH127675 and RS-2021-KH111831) on April 1, 2022. Recruitment for the registry commenced on May 22, 2024, and is scheduled to end on November 30, 2026. As of November 13, 2024, a total of 12 participants have been randomized: 4 participants from Kyung Hee University Korean Medicine Hospital, 1 participant from Dongguk University Ilsan Korean Medicine Hospital, and 7 participants from Wonkwang University Gwangju Korean Medicine Hospital. To date, no data analysis has been conducted.

## Discussion

The potential application of HH333 in ischemic stroke caused by small-vessel disease has been consistently raised. HH333 is Hwangryunhaedoktang (HHT, Orengedokuto, in Japanese; Huanglian Jiedu Tang, in Chinese) combined with *Rhei rhizoma*, with HHT being the main component of HH333. Network pharmacology analysis of HHT to explore its potential applications in brain diseases predicted that it could be used for ischemic and degenerative brain diseases such as ischemic stroke, vascular dementia, and Alzheimer disease [41]. A review of the efficacy and safety of herbal medicine treatments for patients with acute ischemic stroke reported that the combination of HHT and conventional therapies was shown to significantly improve neurological deficits [42]. *Rhei rhizoma*, another component of HH333, also significantly improved neurological deficits and activities of daily living in a review study of herbal medicine containing *Rhei rhizoma* for acute ischemic stroke [43]. Furthermore, previous experimental and clinical studies have demonstrated HH333’s antihyperlipidemic [14], antihypertensive [13], vascular endothelial cell-enhancing [11,12], anti-atherosclerotic [16], cerebral blood flow-enhancing [15], and neuroprotective effects in models of ischemic stroke [44,45], hypoxia [46], and neurodegenerative neurological diseases [47], suggesting that HH333 has the potential to modulate risk factors for ischemic stroke.

Previous retrospective studies of HH333’s effectiveness in inhibiting ischemic stroke recurrence reported that the combination of HH333 and conventional ischemic stroke recurrence prevention therapy was superior to conventional therapy alone [18-20]. Specifically, the effect was the best in small-vessel disease among the Trial of Org 10172 in Acute Stroke Treatment (TOAST) classification of ischemic stroke [20], and the mechanism was proposed to be due to the biochemical effect of HH333 on microangiopathy by acting as an antiapoptotic and cell migration–inducing agent [12].

Most patients with stroke have a multimorbidity status due to various risk factors such as high blood pressure, diabetes, and hyperlipidemia, and controlling risk factors is crucial, making them susceptible to polypharmacy [48]. In a study of the Scottish general population, patients with stroke were more than twice as likely to have multimorbidity compared to patients without stroke, with 12.6% of patients with stroke taking 11 or more medications compared to only 1.5% of patients without stroke [49]. Patients with stroke taking more than 5 or 6 medications are at increased risk for adverse events and falls [50,51], which can severely impact rehabilitation outcomes [48]. Since HH333 has effects on improving stroke risk factors [17], it is expected to complement or replace stroke risk factor therapies, thereby reducing medication use.

Although most herbal medicines are safe and rarely cause liver and kidney injury, some herbal medicines occasionally cause liver injury, and *Scutellariae radix*, a component herbal medicine of HH333, is the main causal herbal medicine responsible for herb-induced liver injury with about 1% of incidence [52]. However, there have been no studies on the dose-dependent toxicity of *Scutellariae radix*, but previous *in vivo* studies have reported no toxicity at a dose of 2000 mg/kg [53,54], which is a much higher dose than the *Scutellariae radix* dose of HH333. In addition, in a 656-patient safety study, no liver or kidney injury was reported with HH333, and mild adverse events of gastrointestinal symptoms, headache, insomnia, chest discomfort, fatigue, and thirst occurred in 2% of patients, with no SAEs [25]. No adverse events were reported in 771 patients in a retrospective cohort study of HH333 published to date [18-20,55]. Therefore, HH333 is expected to be safe with no adverse events, including liver or kidney injury.

Based on this evidence, the Korean Medicine Clinical Practice Guideline for Stroke, compiled by the Korean Ministry of Health and Welfare, recommends that HHT, the main component of HH333, may be considered for the treatment of ischemic stroke to improve neurological disability and prevent recurrence [56]. However, the recommendation is graded as C/Low because clinical studies of HH333 have only been conducted on hypertension and hyperlipidemia, which are risk factors for ischemic stroke, and experimental studies, literature reviews, or retrospective cohort studies on ischemic stroke, which are not sufficient for high quality. In modern clinical research, RCTs represent the highest level of evidence in evidence-based medicine, and significant results from RCTs are more conclusive than other types of clinical research information [57]. Therefore, in order to realize the possibility of HH333 as a therapeutic and recurrent prevention agent for ischemic stroke by improving the quality of evidence, we present a double-blind RCT compared with a placebo to evaluate the clinical effectiveness and safety of HH333 for ischemic stroke in patients with ischemia induced by small-vessel disease.

A limitation of this protocol is that it excluded patients at high risk of bleeding, such as those taking anticoagulants. This exclusion was made because it was important to exclude any factors that could influence bleeding propensity in this study, but it may limit the generalizability of the results.

We hypothesized that HH333 would be effective and safe in preventing recurrent ischemic stroke and improving neurological symptoms in patients with small-vessel disease induced by ischemic stroke. This clinical study will generate data on the therapeutic efficacy and safety of HH333 in ischemic stroke. If the results of this study prove the efficacy and safety of HH333, they will serve as a basis for further use of HH333 with ischemic stroke and will also contribute to the development of novel alternatives for the treatment and prevention of ischemic stroke.

## Appendix

Multimedia Appendix 1 Research consent form.

Multimedia Appendix 2 SPIRIT (Standard Protocol Items: Recommendations for Interventional Trials) checklist.

## Abbreviations

FAS: Fatigue Assessment Scale
FSS: Fatigue Severity Scale
HHT: Hwangryunhaedoktang
K-mBI: Korean Modified Barthel Index
K-MoCA: Korean Montreal Cognitive Assessment
K-NIHSS: Korean National Institutes of Health Stroke Scale
K-PHQ-9: Korean Patient Health Questionnaire
mRS: modified Rankin Scale
RCT: randomized controlled trial
SAE: serious adverse event
SPIRIT: Standard Protocol Items: Recommendations for Interventional Trials
TOAST: Trial of Org 10172 in Acute Stroke Treatment

## Data Availability

Data sharing does not apply to this protocol, as datasets have neither been collected nor analyzed.

## References

[1] Skajaa N, Adelborg K, Horváth-Puhó E, Rothman KJ, Henderson VW, Thygesen LC, Sørensen HT (2022). Risks of stroke recurrence and mortality after first and recurrent strokes in Denmark: a nationwide registry study. Neurology.

[2] Chung JY, Lee BN, Kim YS, Shin B, Kang HG (2023). Sex differences and risk factors in recurrent ischemic stroke. Front Neurol.

[3] Bangad A, Abbasi M, de Havenon A (2023). Secondary ischemic stroke prevention. Neurotherapeutics.

[4] Engel-Nitz NM, Sander SD, Harley C, Rey GG, Shah H (2010). Costs and outcomes of noncardioembolic ischemic stroke in a managed care population. Vasc Health Risk Manag.

[5] Kleindorfer DO, Towfighi A, Chaturvedi S, Cockroft KM, Gutierrez J, Lombardi-Hill D, Kamel H, Kernan WN, Kittner SJ, Leira EC, Lennon O, Meschia JF, Nguyen TN, Pollak PM, Santangeli P, Sharrief AZ, Smith SC, Turan TN, Williams LS (2021). 2021 guideline for the prevention of stroke in patients with stroke and transient ischemic attack: a guideline from the American Heart Association/American Stroke Association. Stroke.

[6] Kim BJ, Kim JS (2014). Ischemic stroke subtype classification: an asian viewpoint. J Stroke.

[7] Hilal S, Mok V, Youn YC, Wong A, Ikram MK, Chen CL (2017). Prevalence, risk factors and consequences of cerebral small vessel diseases: data from three Asian countries. J Neurol Neurosurg Psychiatry.

[8] Litak J, Mazurek M, Kulesza B, Szmygin P, Litak J, Kamieniak P, Grochowski C (2020). Cerebral small vessel disease. Int J Mol Sci.

[9] Kim YS, Jung EA, Shin JE, Chang JC, Yang HK, Kim NJ, Cho KH, Bae HS, Moon SK, Kim DH (2002). Daio-orengedokuto inhibits HMG-CoA reductase and pancreatic lipase. Biol Pharm Bull.

[10] Cho KH, Kim YS, Bae HS, Moon SK, Jung WS, Park EK, Kim DH (2004). Inhibitory effect of chunghyuldan in prostaglandin E2 and nitric oxide biosynthesis of lipopolysaccharide-induced RAW 264.7 cells. Biol Pharm Bull.

[11] Park SU, Jung WS, Moon SK, Ko CN, Cho KH, Kim YS, Bae HS, Chi SG (2005). Chunghyuldan activates NOS mRNA expression and suppresses VCAM-1 mRNA expression in human endothelial cells. Can J Physiol Pharmacol.

[12] Cho KH, Jung WS, Park SU, Moon SK, Ko CN, Ku S, Chi SG, Park H (2004). Daio-orengedokudo works as a cell-proliferating compound in endothelial cells. Can J Physiol Pharmacol.

[13] Yun SP, Jung WS, Park SU, Moon SK, Ko CN, Cho KH, Kim YS, Bae HS (2005). Anti-hypertensive effect of chunghyul-dan (qingxue-dan) on stroke patients with essential hypertension. Am J Chin Med.

[14] Cho KH, Kang HS, Jung WS, Park SU, Moon SK (2005). Efficacy and safety of chunghyul-dan (qingwie-dan) in patients with hypercholesterolemia. Am J Chin Med.

[15] Jin C, Moon SK, Cho SY, Park SU, Jung WS, Park JM, Ko CN, Cho KH, Kwon S (2017). The effect of Chunghyul-dan on hyperventilation-induced carbon dioxide reactivity of the middle cerebral artery in normal subjects: a dose-dependent study. Evid Based Complement Alternat Med.

[16] Park SU, Jung WS, Moon SK, Ko CN, Cho KH, Kim YS, Bae HS (2006). Chunghyul-dan (Qingxie-dan) improves arterial stiffness in patients with increased baPWV. Am J Chin Med.

[17] Jung WS, Kwon S, Cho SY, Park SU, Moon SK, Park JM, Ko CN, Cho KH (2016). The effects of Chunghyul-dan (A Korean Medicine Herbal Complex) on cardiovascular and cerebrovascular diseases: a narrative review. Evid Based Complement Alternat Med.

[18] Cho K, Noh K, Jung W, Park S, Moon S, Park J, Ko C, Kim Y, Bae H (2008). A preliminary study on the inhibitory effect of Chunghyul-dan on stroke recurrence in patients with small vessel disease. Neurol Res.

[19] Jung WS, Min IK, Jin C, Park JY, Kim HG, Kwak Y, Kim KW, Cho SY, Park SU, Moon SK, Park JM, Ko CN, Cho KH, Kwon S (2018). Inhibitory effect of chunghyul-dan on stroke recurrence in small vessel disease patients: a 5-Year observational study. J Evid Based Integr Med.

[20] Lee H, Kwon S, Cho SY, Park SU, Jung WS, Moon SK, Park JM, Ko CN, Jang HJ, Cho KH (2023). Effect of an herbal medicine, Chunghyul-dan, on prevention of recurrence in patients with ischemic stroke: A retrospective cohort study. Medicine (Baltimore).

[21] The Salt Collaborative Group (1991). Swedish aspirin low-dose trial (SALT) of 75 mg aspirin as secondary prophylaxis after cerebrovascular ischaemic events. Lancet.

[22] Diener H, Cunha L, Forbes C, Sivenius J, Smets P, Lowenthal A (1996). European stroke prevention study. 2. Dipyridamole and acetylsalicylic acid in the secondary prevention of stroke. J Neurol Sci.

[23] Diener H, Bogousslavsky J, Brass LM, Cimminiello C, Csiba L, Kaste M, Leys D, Matias-Guiu J, Rupprecht H, MATCH investigators (2004). Aspirin and clopidogrel compared with clopidogrel alone after recent ischaemic stroke or transient ischaemic attack in high-risk patients (MATCH): randomised, double-blind, placebo-controlled trial. Lancet.

[24] Shinohara Y, Katayama Y, Uchiyama S, Yamaguchi T, Handa S, Matsuoka K, Ohashi Y, Tanahashi N, Yamamoto H, Genka C, Kitagawa Y, Kusuoka H, Nishimaru K, Tsushima M, Koretsune Y, Sawada T, Hamada C, CSPS 2 group (2010). Cilostazol for prevention of secondary stroke (CSPS 2): an aspirin-controlled, double-blind, randomised non-inferiority trial. Lancet Neurol.

[25] Cho K, Jung W, Park S, Moon S, Kim Y, Hae H (2003). Clinical assessment on the safety of chunghyul-dan (Qingwie-dan). J Korean Oriental Med.

[26] Krupp LB, LaRocca NG, Muir-Nash J, Steinberg AD (1989). The fatigue severity scale. Application to patients with multiple sclerosis and systemic lupus erythematosus. Arch Neurol.

[27] Michielsen HJ, De Vries J, Van Heck GL (2003). Psychometric qualities of a brief self-rated fatigue measure: the Fatigue Assessment Scale. J Psychosom Res.

[28] Kroenke K, Spitzer RL, Williams JBW (2001). The PHQ-9: validity of a brief depression severity measure. J Gen Intern Med.

[29] Choi HS, Choi JH, Park KH, Joo KJ, Ga H, Ko HJ, Kim SR (2007). Standardization of the Korean version of patient health questionnaire-9 as a screening instrument for major depressive disorder. J Korean Acad Fam Med.

[30] Kim HJ, Bae HS, Park SU, Moon SK, Park JM, Jung WS (2011). Clinical approach to the standardization of oriental medical diagnostic pattern identification in stroke patients. Evid Based Complement Alternat Med.

[31] Jung W, Cho S, Park S, Moon S, Park J, Ko C, Cho K, Kwon S (2019). Development of standardized predictive models for traditional Korean medical diagnostic pattern identification in stroke subjects: a hospital-based multi-center trial. J Korean Med.

[32] Goldstein LB, Bertels C, Davis JN (1989). Interrater reliability of the NIH stroke scale. Arch Neurol.

[33] Oh MS, Yu KH, Lee JH, Jung SJ, Ko IS, Shin JH, Cho SJ, Choi HC, Kim HH, Lee BC (2012). Validity and reliability of a korean version of the national institutes of health stroke scale. J Clin Neurol.

[34] van Swieten JC, Koudstaal PJ, Visser MC, Schouten HJ, van Gijn J (1988). Interobserver agreement for the assessment of handicap in stroke patients. Stroke.

[35] Kim K (2012). Risk factors and clinical outcome of ischemic stroke in the very elderly. J Neurocrit Care.

[36] Shah S, Vanclay F, Cooper B (1989). Improving the sensitivity of the barthel index for stroke rehabilitation. J Clin Epidemiol.

[37] Jung HY, Park BK, Shin HS, Kang YK, Pyun SB, Paik NJ, Kim SH, Kim TH (2007). Development of the Korean version of modified barthel index (K-MBI): multi-center study for subjects with stroke. J Korean Acad Rehabil Med.

[38] Petersen RC (2004). Mild cognitive impairment as a diagnostic entity. J Intern Med.

[39] Kang Y, Park J, Yu KH, Lee BC (2009). A reliability, validity, and normative study of the Korean-montreal cognitive assessment(K-MoCA) as an instrument for screening of vascular cognitive impairment(VCI). Kor J Clin Psychol.

[40] Chan A, Tetzlaff JM, Gøtzsche PC, Altman DG, Mann H, Berlin JA, Dickersin K, Hróbjartsson A, Schulz KF, Parulekar WR, Krleza-Jeric K, Laupacis A, Moher D (2013). SPIRIT 2013 explanation and elaboration: guidance for protocols of clinical trials. BMJ.

[41] Lee SE, Lim JY, Chung BW, Lee B, Lim JH, Cho S (2020). Network pharmacological analysis for exploration of the potential application of Hwangryunhaedok-tang for brain diseases. Herb Formula Sci.

[42] Han CH, Kim M, Cho SY, Jung WS, Moon SK, Park JM, Ko CN, Cho KH, Kwon S (2018). Adjunctive herbal medicine treatment for patients with acute ischemic stroke: a systematic review and meta-analysis. Complement Ther Clin Pract.

[43] Lu L, Li H, Fu D, Zheng G, Fan J (2014). Rhubarb root and rhizome-based Chinese herbal prescriptions for acute ischemic stroke: a systematic review and meta-analysis. Complement Ther Med.

[44] Park TH, Lee HG, Cho SY, Park SU, Jung WS, Park JM, Ko CN, Cho KH, Kwon S, Moon SK (2023). A comparative study on the neuroprotective effect of geopung-chunghyuldan on in vitro oxygen-glucose deprivation and in vivo permanent middle cerebral artery occlusion models. Pharmaceuticals (Basel).

[45] Lee HG, Kwon S, Moon SK, Cho SY, Park SU, Jung WS, Park JM, Ko CN, Cho KH (2023). Neuroprotective effects of geopung-chunghyuldan based on its salvianolic acid B content using an in vivo stroke model. Curr Issues Mol Biol.

[46] Ko CN, Park IS, Park SU, Jung WS, Moon SK, Park JM, Kang C, Cho KH (2013). Neuroprotective effect of chunghyuldan (Qing Xue Dan) on hypoxia-reoxygenation induced damage of neuroblastoma 2a cell lines. Chin J Integr Med.

[47] Kim HG, Ju MS, Kim D, Hong J, Cho S, Cho K, Park W, Lee EH, Kim SY, Oh MS (2010). Protective effects of chunghyuldan against ROS-mediated neuronal cell death in models of Parkinson's disease. Basic Clin Pharmacol Toxicol.

[48] Kose E, Toyoshima M, Okazoe S, Oka R, Shiratsuchi Y, Hayashi H (2017). The relationship between polypharmacy and recovery of activities of daily living among convalescent stroke patients: a propensity score-matched analysis. Eur Geriatr Med.

[49] Gallacher KI, Batty G, McLean G, Mercer SW, Guthrie B, May CR, Langhorne P, Mair FS (2014). Stroke, multimorbidity and polypharmacy in a nationally representative sample of 1,424,378 patients in Scotland: implications for treatment burden. BMC Med.

[50] Kojima T, Akishita M, Kameyama Y, Yamaguchi K, Yamamoto H, Eto M, Ouchi Y (2012). High risk of adverse drug reactions in elderly patients taking six or more drugs: analysis of inpatient database. Geriatr Gerontol Int.

[51] Kojima T, Akishita M, Nakamura T, Nomura K, Ogawa S, Iijima K, Eto M, Ouchi Y (2012). Polypharmacy as a risk for fall occurrence in geriatric outpatients. Geriatr Gerontol Int.

[52] Mantani N (2023). Liver injury related to Japanese herbal medicines: clinical features and diagnosis. Explor Dig Dis.

[53] Kim MS, Ham SH, Kim JH, Shin JE, Oh JO, Kim TW, Yun HI, Lim JH, Jang BS, Cho JH (2012). Single-dose oral toxicity of fermented scutellariae radix extract in rats and dogs. Toxicol Res.

[54] Lee JW, Jung YS, Kim JD, Choi HY (2013). Mouse single oral dose toxicity test of scutellariae radix aqueous extracts. J Int Korean Med.

[55] Cho KH, Ji NG, Jung WS, Moon SK, Bae HS (2005). Chunghyul-dan for the prevention of stroke progression in silent brain infarction. J Korean Med.

[56] The Society of Stroke on Korean Medicine (2021). The Korean Medicine Clinical Practice Guideline for Stroke. National Institute for Korean Medicine Development.

[57] Stanley K (2007). Design of randomized controlled trials. Circulation.

