# Supplementary material for: Clinical Efficacy and Safety of the Herbal Prescription, HH333, in Preventing Recurrent Stroke in Patients With Ischemic Stroke Induced by Small-Vessel Disease: Protocol for Multicenter, Double-Blind, Randomized, Prospective, Pilot Clinical Trial
Source: JMIR Res Protoc. 2025 May 13;14:e70953. doi: 10.2196/70953 (PMC12117272; doi:10.2196/70953)
Supplement: Multimedia Appendix 1 [file resprot_v14i1e70953_app1.docx]

**RESEARCH CONSENT FORM**

| Research project | Clinical Efficacy and Safety of the Herbal Prescription, HH333 in Preventing Recurrent Stroke in Patients with Ischemic Stroke induced by Small Vessel Disease: A Study Protocol from a Multicenter, Double-blind, Randomized, Prospective, Pilot Clinical Trial | |
| --- | --- | --- |
| Clinical trial institution | Kyung Hee University Korean Medicine Hospital, Dongguk University Ilsan Korean Medicine Hospital, Wonkwang University Gwangju Korean Medicine Hospital |  |
| Principal Investigator | Seungwon Kwon |  |
| Supporting Organization | Korea Health Industry Development Institute |  |
| Institutional Review Board | Kyung Hee University Korean Medicine Hospital Institutional Review Board | |

Please read the following carefully to ensure that you are fully informed by the researcher and

check the box if you are willing to participate.

1. I have read the description of this study and discussed it with the researcher. **□**
2. I have been informed of the risks and benefits of this study and have received satisfactory answers to my questions. **□**
3. I have been fully informed by the researcher of the benefits and risks associated with this study, and I have been informed that I may request a full explanation of this matter from the researcher at any time. **□**
4. I voluntarily consent to participate in this study. **□**
5. I understand that I may withdraw from participation in this study at any time and that this decision will not harm me in any way. **□**
6. 6. I consent to the researcher collecting and processing my information obtained in this study within the scope permitted by current laws and the regulations of the Institutional Review Board. **□**
7. I agree that after the study is completed, the data of the research subjects may be registered in a public database with their personally identifiable information anonymized. □

**<Personal information collection and use details>**

| Personal information items | Purpose of collection | Retention period |
| --- | --- | --- |
| - Name, gender, date of birth | Clinical efficacy and safety of the herbal prescription, HH333 in preventing recurrent stroke in patients with ischemic stroke induced by small vessel disease | 3 years after the end of the study |

**※** **You have the right to refuse to provide personal information.**

**☞** **Do you agree to the collection and use of personal information as above? (□ Yes, □ No)**

**<** **Collection and use of sensitive information >**

| Sensitive information items | Purpose of collection | Retention period |
| --- | --- | --- |
| - Medical records (medical history/comorbidities, test results, questionnaires, concurrent medications, physical examination, physical measurements, disease progress/condition observation, vital signs) - Health-related behavior information (physical activity, smoking, drinking status: past and current history, amount of smoking and drinking) | Clinical efficacy and safety of the herbal prescription, HH333 in preventing recurrent stroke in patients with ischemic stroke induced by small vessel disease | 3 years after the end of the study |

**※** **You have the right to refuse to provide sensitive information.**

**☞** **Do you agree to the collection and use of personal information as above? (□ Yes, □ No)**

**<Provision and entrustment of personal information to third parties>**

| Recipient | Items provided | Purpose of collection | Retention period |
| --- | --- | --- | --- |
| Korea Health Industry Development Institute | - Gender, date of birth - Medical records (medical history/comorbidities, test results, questionnaires, concurrent medications, physical examination, physical measurements, disease progress/condition observation, vital signs) - Health-related behavior information (physical activity, smoking, drinking status: past and current history, amount of smoking and drinking) | Clinical efficacy and safety of the herbal prescription, HH333 in preventing recurrent stroke in patients with ischemic stroke induced by small vessel disease | 3 years after the end of the study |

**※You have the right to refuse provision and entrustment to third parties.**

**☞** **Do you agree to the collection and use of personal information as above? (□ Yes, □ No)**

1. I agree that when the research team and the authorized representative conduct research or manage the results, and that the monitoring agent, inspection agent, Institutional Review Board, Minister of Health and Welfare, and Minister of the Ministry of Food and Drug Safety may access my medical records to verify the research implementation procedure and data quality in accordance with relevant laws and regulations, within the scope that the confidentiality of my personal information is protected, and that access to such data is permitted with a consent form signed by me or my agent. □
2. My signature indicates that I have received a copy of this consent form, and I will keep a copy until my participation in the study is complete. □

**I fully understand the consent form and agree to the above by signing below.**

| Research subject | Name: | Signature: | Date: / / |
| --- | --- | --- | --- |
| Legal representative (if necessary) | Name: | Signature: | Date: / / |
|  | * Relationship to research subject: (parent, spouse, guardian) | | |
|  | * Reason: | | |
| Observer (if necessary) | Name: | Signature: | Date: / / |
| Researcher | Name: | Signature: | Date: / / |
